# Effect of Probiotics *Lactobacillus rhamnosus* and *Lactobacillus plantarum* on Caries and Periodontal Diseases: A Systematic Review

**DOI:** 10.3390/dj12040102

**Published:** 2024-04-10

**Authors:** Silvia D’Agostino, Giulia Valentini, Francesca Iarussi, Marco Dolci

**Affiliations:** 1 Complex Unit of Odontostomatology, Interdisciplinary Department of Medicine, University A. Moro, 70124 Bari, Italy; 2 Department of Medical, Oral and Biotechnological Sciences, University G. d’Annunzio, 66100 Chieti, Italy

**Keywords:** caries, periodontitis, probiotics, *Lactobacillus rhamnosus*, *Lactobacillus plantarum*

## Abstract

Caries and periodontitis are the most prevalent oral diseases worldwide. Major factors contributing to the development of these oral conditions include poor oral hygiene, dental biofilm formation, high carbohydrates diet, smoking, other systemic diseases, and genetic factors. Various preventive measures have been established to mitigate the risk of caries and periodontal disease development. The present review aims to discuss the role of the probiotics *Lactobacillus rhamnosus* and *Lactobacillus plantarum* in the prevention and treatment of caries and periodontal diseases. The study was conducted in accordance with PRISMA guidelines and was registered on PROSPERO. The search involved PubMed, Web of Science, and Scopus and considered the PICO format. Studies were screened by two reviewers independently, and disagreements were solved by consensus with a third reviewer. Data extraction included details about the type of probiotics, strains, and purpose of administration. A total of 15 RCTs were included, of which just 1 was about tooth cavities. Overall, 87% of the included studies were good-quality papers regarding the Jadad Scale. Several studies agreed on the potential of probiotics *L. rhamnosus* and *L. plantarum*, both alone and combined, to prevent and improve clinical outcomes in caries and periodontal treatments, weaker evidence is provided for the microbiological benefit.

## 1. Introduction

The last World Health Organization (WHO) Global Oral Health Status Report in 2022 stated that an estimated two billion people worldwide suffer from caries of permanent dentition and assumed that over 19% of adults globally suffer from severe periodontal disorders, amounting to over 1 billion cases worldwide. The biggest risk factor for periodontal disease and caries is poor dental hygiene [1].

Dental caries, a chronic and multifaceted condition, manifests as the degradation and demineralization of tooth enamel and dentin by cariogenic bacteria. The oral cavity is inhabited by several microbial strains and when a disruption of this equilibrium happens, it represents a possible causative factor in the development of dental caries. Various factors contribute to caries development, including poor oral hygiene, a diet rich in cariogenic carbohydrates, the buildup of dental plaque, the presence of cavity-causing bacteria, and genetic predisposition [2,3,4,5].

The formation of dental caries is initiated by the acidic byproducts of carbohydrate metabolism produced by resident oral microbes. These acids lower the pH of the oral environment, leading to the demineralization of tooth enamel and dentin. Prolonged demineralization eventually results in the formation of cavities on tooth surfaces [6].

However, multiple demineralization/remineralization events occur during the day, and if demineralization is a predominant process, this may lead to caries development. The remineralization of demineralized tooth surfaces can be facilitated by the use of fluoride supplements. Fluoride ions can bind to tooth enamel and dentin, making them more resistant to acid attacks and promoting remineralization [7]. A disruption in the delicate balance of the oral microbial community can also trigger cavitation. The overgrowth of certain bacterial species, particularly those that produce glucansucrases, can lead to the release of enzymes that further demineralize tooth surfaces and contribute to the formation of cavities [8].

Periodontal diseases, such as gingivitis, periodontitis, peri-implant mucositis, and peri-implantitis, are caused by the complex interplay between pathogenic microorganisms and the host’s defensive mechanisms. This intricate relationship gives rise to a chronic inflammatory response that, if left unchecked, can lead to the destruction of the supporting tissues around teeth, resulting in periodontitis [9,10]. It is well established that mechanical removal of dental plaque is the core of the periodontal therapy; this essential step disrupts the biofilm, the sticky accumulation of bacteria that forms in the supra- and sub-gingival environment, and helps to control periodontal disease and prevent tooth loss [11,12]. While subgingival instrumentation is crucial for periodontal therapy, its effectiveness in eradicating periodontal pathogens is not always guaranteed. This is due to the bacteria’s ability to escape mechanical debridement by burrowing into soft tissues or taking refuge in hard-to-access anatomical structures, such as dentinal tubuli, furcations, or deep infrabony defects [13,14,15,16,17].

The growing interest in probiotics as an alternative adjunctive treatment for periodontitis and a preventive tool for caries has captured the attention of the scientific community, with promising results emerging from recent studies. Probiotics are live microorganisms that, when administered in adequate amounts, confer a health benefit on the host [18]. The elaborate balance of microorganisms that reside within our bodies, collectively known as the microbiota, plays a pivotal role in regulating our physiological, immunological, and psychological well-being. Generally, a harmonious coexistence between the microbiota and the host system is essential for optimal health, particularly in maintaining efficient metabolism and a robust immune response. However, disturbances to this delicate equilibrium caused by external factors such as pathogenic infections or immunosuppressant medications, and/or internal factors like autoimmune disorders, can lead to a range of health issues, from mild to severe. Age, mode of delivery, genetic makeup, dietary patterns, antibiotic use, and the consumption of probiotics and prebiotics are all significant factors that influence the composition and function of the gut microbiota, which, in turn, can impact overall health [19]. *Lactobacillus rhamnosus* and *Lactobacillus plantarum* have long been used in caries prevention [20,21] and in supportive periodontal therapy [22,23]. These beneficial bacteria effectively inhibit the growth of oral pathogens, including *Streptococcus mutans* and *Porphyromonas gingivalis*. Notably, *L. plantarum* combats *Aggregatibacter actinomycetemcomitans* and *Prevotella intermedia*. Moreover, both strains exhibit remarkable resilience to acidic environments and can effectively colonize the oral cavity [21,22,23]. As stated by Yang Y. et al. [21], *L. plantarum* shows promise as a probiotic for oral health due to its low potential to cause cavities result of a low adhesion ratio to hard tissue and its broad-spectrum inhibitory activity against harmful bacteria. Mendi A. et al. [22] pointed out that *L. rhamnosus* could prevent *P. gingivalis* from suppressing the production of CXCL8, a key immune molecule, through co-aggregation with this periodontal pathogen, competing for adhesion sites on oral surfaces, and triggering TLR-mediated immune responses.

The aim of this review is to explore the effect of *L. rhamnosus* and *L. plantarum* on oral conditions such as caries and periodontal diseases.

## 2. Materials and Methods

A systematic review was conducted using the Preferred Reporting Items for Systematic Reviews and Meta-Analyses (PRISMA) guidelines for systematic reviews and meta-analysis [24] and registered on PROSPERO—International prospective register of systematic reviews—with the ID code CRD42023453915.

### 2.1. Literature Search

The objective of the literature browsing was to define pertinent studies analyzing the possible effect of the oral probiotics *L. rhamnosus* and *L. plantarum* on oral health in the last ten years. An exhaustive search of PubMed, Web Of Science, and Scopus, using the Patient/Population/Problem, Intervention, Comparison, and Outcome (PICO) format, was conducted.

- Population: humans of all ages;
- Intervention: adjunctive option in non-surgical periodontal therapy and caries;
- Comparator: healthy adults/adolescents/children;
- Outcomes: possible effects of orally administered *L. rhamnosus* and *L. plantarum* (alone or in several combinations) on periodontitis/gingivitis/peri-implant mucositis and tooth decay.

The following MeSH (Medical Subject Headings) were used: *Lactobacillus rhamnosus*; *Lactobacillus plantarum*; AND periodontitis, AND gingivitis, AND mucositis, AND dental caries. The search time period started on 20 November 2023 and ended on 20 December 2023.

### 2.2. Eligibility Criteria

The inclusion criteria were as follows: all in vivo studies on humans analyzing the effects of *L. rhamnosus* and *L. plantarum* in humans, in the English language.

The following served as exclusion criteria: research about other strains of *Lactobacillus* in the same oral conditions; papers about oral probiotics and non-oral environments (e.g., gut microbiota, inflammatory bowel disease, immunological responses, vaccines); systematic reviews; metanalyses; editorials; abstracts; book chapters; papers not in English.

### 2.3. Data Extraction

Studies were appraised by two reviewers independently (S.D., G.V.), and a matrix of relevant data was produced. In cases of reviewer disagreement, consensus was sought, and if necessary, a third reviewer provided a final decision (M.D.). Data extraction included general details corresponding to the properties of the studies (e.g., author, year of publication, sources of funding) and specific details about the type of probiotics, strains, and purpose of administration.

### 2.4. Quality Assessment

As shown in Table 1, a data quality assessment of the included studies was evaluated using the Jadad Scale (also known as the Oxford quality scoring system) [25]. It constitutes five total points: two points regarding the randomization, two points applicable to blinding, and one point addressing the dropout. Concerning randomization and blinding, when there are generic considerations without any definition about randomization or blinding, one point each is assigned to each specific category. When there is a characterization of the appropriate method, one point is augmented to each respective category. When the description strategy is inappropriate, one point is removed from each respective category. For dropout, when the number of dropouts for each subject group and the reasons for dropout are indicated, one point is added. Even if there is no dropout, this fact should be stated. When the total for the Jadad score is ≥3 points, the study is considered to “high-quality”; when ≤2 points, it is considered “low-quality”. For studies in which double blinding is not impossible, a study with a total score ≥2 points is considered “high-quality”. The Jadad scale is convenient because of its clarity, but it does not include an appraisal item for allocation concealment. However, there is not any standardized assessment tool to carry out a quality assessment of an RCT.

All included studies are proposed in Table 2 including authors, year, population, age, main characteristic, *Lactobacillus* strain, time, and purpose of the administration. A brief narrative summary is displayed in Table A1.

## 3. Results

### 3.1. Study Selection

**Figure 1:**
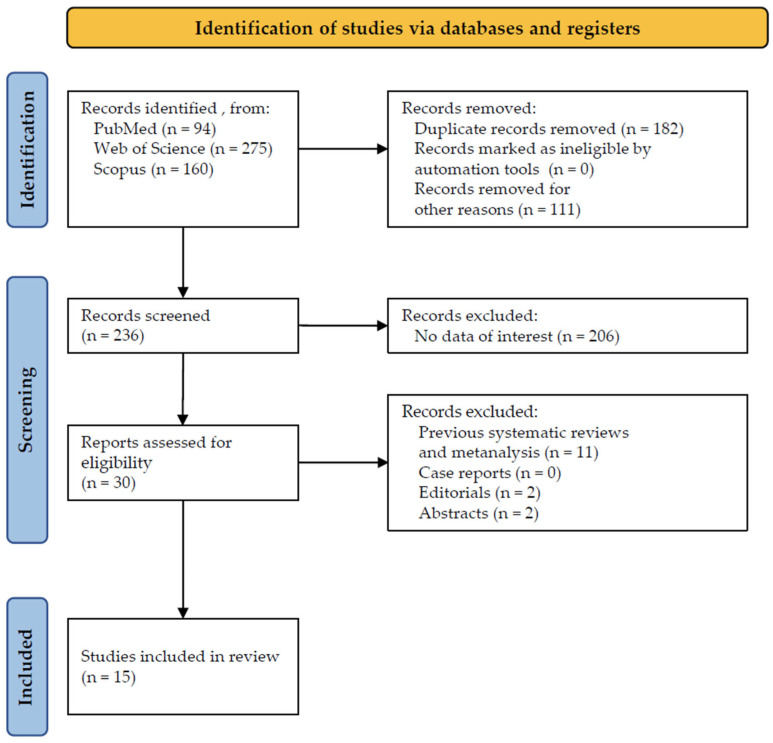
PRISMA flowchart.

The starting search supplied a total of 529 studies: 94 from PubMed, 275 from Web of Science, and 160 from Scopus. No studies were deleted due to being ineligible by automation tools, while 182 studies were removed because of duplication. Overall, 111 studies were removed for other reasons, for example, for their employment in other districts (gut, immune system, cardiovascular system). A total of 236 studies accessed the screening phase, and a total of 206 studies were withdrawn because they failed to demonstrate any data of interest, for example, because they were conducted in vitro or in animals. Eligibility was assigned to 30 studies based on their abstracts, from which 11 were erased for being systematic reviews, 2 for being abstracts, and 2 for being editorials. Finally, a total of 15 studies were incorporated for the inclusion phase (Figure 1) and analyzed according to their full text.

### 3.2. Detailed Results

Regarding the population age, 66.6% (10/15) of studies refer to adults, 6.7% (1/15) refer to adolescents, 6.7% (1/15) refer to children, and, finally, 20% (3/15) did not specify the age of the sample. The most investigated characteristic of the samples was periodontitis, in 40% of studies (6/15). Healthy patients were studied in 33.4% (5/15) of manuscripts. People with intellectual diseases represented 13.3% (2/15) and, finally, peri-implant mucositis were investigated in 13.3% (2/15) of cases. *L. rhamnosus* was investigated alone in 33.3% (5/15) of the studies and in association with other microorganisms in 40% (6/15) of the studies. *L. plantarum* was investigated alone in 6.7% (1/15) of the studies and in association in 20% (3/15) of the studies. No study explored the association between *L. rhamnosus* and *L. plantarum*. Considering the time of administration, 53.3% (8/15) presented 3 months of treatment, 26.7% (4/15) had 1 month, 13.3% (2/15) had 1.5 months, and just in 6.7% (1/15) there were 10–12 months of treatment. Overall, 53.3% (8/15) of the studies concerned periodontitis, 26.7% (4/15) of the studies concerned gingivitis, 13.3% (2/15) of studies concerned peri-implant mucositis, and, finally, 6.7% (1/15) investigated caries. Regarding the form of the probiotics used, 20% (3/15) employed lozenges, 20% (3/15) prescribed sachets, 20% (3/15) utilized tablets, 13.3% (2/15) involved yogurt, 13.3% (2/15) applied gel and lozenges, 6.7% (1/15) suggested mouthwash, and 6.7% (1/15) recommended capsules. In view of these findings, it should be noted that the majority of the studies included analyzed an adult sample, while data about adolescents and children remain weak due to the lack of studies suitable. It is remarkable that more than half of the studies included reported an administration time of at least 3 months, suggesting the hypothesis that shorter administrations could not be useful to effectively modulate the local microbiota. Additionally, the probiotic form—lozenges, sachets, tablets, yogurt, gel, mouthwash, capsules—raises questions about the differences in the gastric absorption of each solution adopted and about the topically absorption for gels and mouthwashes. Our results show that *L. rhamnosus* seems to be the strain used for caries prevention; in particular, a daily intake of lozenges containing *L. rhamnosus* and *L. paracasei*, combined with arginine, may have potential in lowering the rate of new cavities in children aged 5–9 [27]. With reference to periodontal disease, the data display an equal use of both *L. rhamnosus* and *L. plantarum* alone [26,30,32,34,38,39] or in combination with other strains [27,28,29,31,33,35,36,37,40]. As stated by Morales A. et al. [39], *L. rhamnosus* may exert regulatory effects on cytokine production by peripheral blood mononuclear cells exposed to anti-CD3 antibodies, indicating potential immune-boosting properties for all ages. On the other hand, *L. plantarum* was defined by Iwasaki K. et al. [38] as being responsible for an enhanced T-helper1 cell activity and interferon-γ production in healthy adults, suggesting potential benefits for combating periodontitis.

### 3.3. Quality Assessment Results

With reference to the Jadad Scale for the quality assessment, 86.7% (13/15) were good-quality studies, more specifically 13.3% (2/15) had a score of 3, 46.7 (7/15) had a score of 4, and 26.7% (4/15) had a score of 5. Finally, 13.3% (2/15) were low-quality studies; in particular, 1 study had a score of 2, and another had a score of 1. Due to a considerable heterogeneity in collecting data, the use of several probiotics’ associations, employment of different probiotic forms, and time of the treatment, a formal meta-analysis was not carried out. The included studies used different ways to measure the same outcome (e.g., different clinical indexes as Probing Pocket Depth (PPD), Plaque Index (PI), Gingival Index (GI), Bleeding on Probing (BOP), making it difficult to standardize and combine the data meaningfully.

## 4. Discussion

The precise mechanisms by which probiotics exert their beneficial effects in the oral cavity remain to be fully elucidated, but several studies have demonstrated a correlation between probiotic consumption and reduced levels of cariogenic and periodontal pathogens. One proposed mechanism involves competitive exclusion, where probiotics outcompete harmful bacteria for adhesion sites on teeth and mucosal surfaces, effectively preventing their colonization and reducing their overall numbers [41]. Another mechanism involves the production of antimicrobial substances, such as bacteriocins, which directly inhibit the growth and activity of cariogenic and periodontal pathogens. Probiotics may also modulate the host’s immune response, enhancing the body’s ability to combat oral infections and maintain overall oral health [42]. The majority of studies reviewed highlight the ability of probiotics to compete with pathogenic bacteria for adhesion sites and nutrients, effectively displacing and reducing the overall abundance of harmful microorganisms [26,27,28,29,30,31,32]. None of the studies reviewed reported side effects linked with the use of oral probiotics, and only 9 studies described the dropout motivation, in particular, for personal reasons, or the unpleasant taste of the probiotic, neglecting to ingest the probiotic [27,31,38,39,40]. It should be noted that Oda Y. et al. [26] and Yuki et al. [32] referred to the use of oral probiotics based on *L. rhamnosus* in people with intellectual disability with periodontal disease as tools to lower the risk and stop the progression of periodontitis. The two most prominent areas of focus in this systematic review are periodontal disease and dental caries, which are examined independently.

### 4.1. Dental Caries and Probiotics

Dental caries, also identified as tooth decay, is a complex disease arising from a combination of factors, including the oral microbiome, the host’s immune system, and dietary habits. The primary culprit is *Streptococcus mutans*, which thrives on fermentable carbohydrates in the diet and produces acids that dissolves enamel hydroxyapatite [2,5]. The host’s immune system plays a role in limiting bacterial growth and promoting remineralization, but its effectiveness depends on individual factors like saliva composition and salivary flow rate [3]. Despite all these elements, microbiological factors are still the dominant etiology of this multifactorial disease. Just one RCT in this review, by Pørksen CJ. et al. [27], referred to dental caries, and the authors administered lozenges with *L. rhamnosus*, *L. paracasei* and arginine in children aged 5–9 and monitored clinical and radiographical scores for 10–12 months. The authors stated that a daily ingestion of a lozenge containing prebiotic arginine; a further two strains of probiotics presented safe use and statistically significantly reduction in caries increase Authors put forward the idea that the use of a lozenge with arginine and probiotics integrated has a promising potential as a supplementary tool for the future management of caries. However, the study also showed some limitations such as the short observation, the calibration only on caries without mentioning other enamel impairments (MIH, erosion), the examiners not knowing about the motivation of previous filling teeth (prophylactically or therapeutically), and, finally, the authors not recording the accurate time of consumption [27].

### 4.2. Periodontal Disease and Probiotics

A disruption of the normal balance between saprophytic and pathogenic oral microbiota can lead to periodontal disease in susceptible individuals. Treatment for periodontal disease typically involves debridement, which can be surgical or nonsurgical. In some cases, systemic antimicrobials may also be prescribed. However, bacterial resistance to these drugs is increasing, necessitating the development of novel approaches to periodontal health maintenance [9,10]. Ranjith A. et al. [28] tested a mouthwash containing the probiotics *L. rhamnosus*, *L. acidophilus*, *B. longum*, *and S. boulardii* as an additional option for stage II periodontitis, alongside mechanical plaque removal. Despite the promising results in terms of PI, GI, PPD, and CAL, this RCT did not specify the age of the sample and established just 1 month of administration. Morales A. et al. [30] stated that *L. rhamnosus* failed to enhance additional benefits in stage III periodontitis patients, in line with their previous results when L. rhamnosus apported a similar reduction in the local flora and clinical parameters compared to placebo and azithromycin groups [34]. However, it is in contrast with the paper by the same authors where the L. rhamnosus group showed a greater reduction in PPD than the placebo group [39]. Pudgar P. et al. [31] stated that the adjunction of a probiotic based on *L. plantarum* and *L. brevis* to mechanical debridement led to a lower amount of gingival bleeding when compared to the placebo group. In addition to those results, Iwasaki K. et al. [38] proposed that integrating a daily capsule supplemented with *L. plantarum* into a supportive periodontal therapy (SPT) regimen may lead to a significant decrease in PPD for patients.

Gingivitis was explored as well. Alanzi A. et al. [33] assumed that incorporating *L. rhamnosus* and *B. lactis* lozenges alongside regular oral care practices could potentially enhance oral health in adolescents between the ages of 13 and 15; in fact, a statistically significant reduction in GI was seen in the probiotic group as compared to the placebo group. A significant reduction was also noted in the total salivary bacterial counts of the test group. In line with this, Keller MK et al. [35] concluded that tablets with *L. rhamnosus* and *L. curvatus* are able to reduce BOP and the flow of gingival crevicular fluid (GCF), but not microbiological parameters in patients with moderate gingivitis. The effectiveness on clinical parameters and the inefficacy on the microbiological aspects of lozenges with *L. rhamnosus* and *B. lactis* was also supported by Toiviainen A. et al. [40]. In contrast with this, Montenero E. et al. [37] affirmed that integrating *L. plantarum*, *L. brevis*, and *P. acidilactici* into gingivitis treatment regimens can enhance the subgingival microbiota.

Additionally, peri-implant mucositis was investigated. According to Santana SI et al. [29] gels and lozenges incorporating *L. rhamnosus*, *L. paracasei*, and *B. lactis* may offer additional therapeutic effects alongside mechanical debridement in peri-implant mucositis management, considering BOP. Additionally, the test group presented lower levels of interleukin (IL)-1β, IL-6, IL-8, and tumor necrosis factor (TNF)-α than controls at baseline. These results are in contrast with those of Mongardini C. et al. [36], who stated that the addition of *L. plantarum* and *L. brevis* to professionally administered plaque removal did not lead to notable improvements in clinical outcomes (Modified Plaque Index (mPlI) and BOP) for patients with peri-implant mucositis.

Finally, Oda Y. et al. [26] and Yuki et al. [32] highlight the potential of yogurt supplemented with *L. rhamnosus* as a dietary intervention for preventing periodontitis in individuals with intellectual disabilities, and it may be considered as a tool to stop the progression of periodontitis. This group of patients presents an adjunctive risk factor for periodontal diseases, and they may need a customized prophylactical and therapeutical approach.

This review did not identify any studies investigating the combined effects of *L. rhamnosus* and *L. plantarum*, suggesting a potential gap in the interaction of these specific strains of probiotics.

### 4.3. Limitations of the Study

The first obstacle that appeared from our analysis is that several studies administered various probiotic strains at the same time, creating confusion in terms of meaning and difficulty in drawing univocal conclusions. Occasionally, the specific age of the samples is left out, making the pertinence analysis difficult. The presence of heterogeneous samples, different ages, different kinds of administration (mouthwashes, sachets, lozenges, yogurt), different periods of administration are all confounding factors. Additionally, the division of patients into those with periodontitis or peri-implant mucositis and healthy ones (with caries and gingivitis) may be misleading because patients may be otherwise healthy but have an oral disease such as gingivitis or caries, and this does not reflect oral health. Finally, the absence of unequivocal conclusions on the use of a probiotic containing a single strain at a time represents a severe bias and makes comparison between studies unclear.

## 5. Conclusions

With the limitations of the study, probiotics were proposed as a form of bacteriotherapy; however, just one of the included studies explained their use in reducing clinical and radiographical parameters in children with dental caries. Regarding periodontal diseases, clinical parameters seem to be improved by probiotic administration, while microbiological aspects remain unclear. The supply of different strains simultaneously makes the comparison between the RCTs difficult and opens a wide debate. Nevertheless, it is crucial to acknowledge that these diseases are multifactorial in nature, meaning that simply reducing clinical criteria may not guarantee complete disease control. Large-scale, well-designed RCTs with consistent methodologies and extended follow-up periods are necessary to establish the definitive role of probiotics in managing these diseases and their impact on disease prevalence. Additionally, identifying the specific probiotic strains that exhibit efficacy for each infectious oral disease is crucial to determine optimal dosage, treatment duration, and delivery methods. Based on the currently available studies, a probiotic combining *L. rhamnosus* and *L. plantarum* was not tested on caries and periodontal diseases.

## Figures and Tables

**Table 1:** Jadad Scale Critical Appraisal Checklist for Cohort Studies [25]. Q1: Was the study described as randomized? Q2: Was the study described as double blind? Q3: Was there a description of withdrawals and dropouts? The maximum score is 5 points. When the total is ≥3 points, the study is considered to be “high-quality”; when ≤2 points, it is considered “low-quality”.

Authors/Year	Q1	Q2	Q3	Tot
Oda Y. et al., 2023 [26]	1	0	0	1
Pørksen CJ. et al., 2023 [27]	2	2	1	5
Ranjith A et al., 2022 [28]	1	1	0	2
Santana SI et al., 2022 [29]	2	2	0	4
Morales A. et al., 2021 [30]	2	2	0	4
Pudgar P. et al., 2021 [31]	2	2	1	5
Yuki O. et al., 2019 [32]	2	2	0	4
Alanzi A. et al., 2018 [33]	1	2	1	4
Morales A. et al., 2018 [34]	2	2	1	5
Keller MK. et al., 2018 [35]	1	1	1	3
Mongardini C. et al., 2017 [36]	2	2	1	5
Montero E. et al., 2017 [37]	2	1	1	4
Iwasaki K. et al., 2016 [38]	2	1	1	4
Morales A. et al., 2016 [39]	2	1	0	3
Toiviainen A. et al., 2015 [40]	1	2	1	4

**Table 2:** Keys results. N/A, not available.

Authors/Year	Population (Age)/ Characteristics	Microorganism	Time of Administration	Aim
Oda Y. et al., 2023 [26]	n = 41 (N/A)/intellectual disability	*L. rhamnosus*	3 months	Periodontitis
Pørksen CJ. et al., 2023 [27]	n = 343 (5–9)/Healthy	*L. rhamnosus*, *L. paracasei*, arginine	10–12 months	Caries
Ranjith A. et al., 2022 [28]	n = 60 (N/A)/Stage II periodontitis	*L. rhamnosus*, *L. acidophilus*, *B. longum*, *S. boulardii*	1 month	Periodontitis
Santana SI et al., 2022 [29]	n = 36 (N/A)/Peri-implant mucositis	*L. rhamnosus*, *L. paracasei*, *B. lactis*	3 months	Peri-implant mucositis
Morales A. et al., 2021 [30]	n = 47 (≥35)/Stage III periodontitis	*L. rhamnosus*	3 months	Periodontitis
Pudgar P. et al., 2021 [31]	n = 40 (25–80)/Stage III-IV periodontitis	*L. plantarum*, *L. brevis*	3 months	Periodontitis
Yuki O. et al., 2019 [32]	n = 23 (20–45)/intellectual disability	*L. rhamnosus*	3 months	Periodontitis
Alanzi A. et al., 2018 [33]	n = 101 (13–15)/Healthy	*L. rhamnosus*, *B. lactis*	1 month	Gingivitis
Morales A. et al., 2018 [34]	n = 47 (≥35)/Stage III periodontitis	*L. rhamnosus*	3 months	Periodontitis
Keller MK. et al., 2018 [35]	n = 47 (≥25)/Healthy	*L. rhamnosus*, *L. curvatus*	1 month	Gingivitis
Mongardini C. et al., 2017 [36]	n = 20(≥46)/Peri-implant mucositis	*L. plantarum*, *L. brevis*	1.5 months	Peri-implant mucositis
Montero E. et al., 2017 [37]	n = 59(18–55)/Healthy	*L. plantarum*, *L. brevis*, *P. acidilactici*	1.5 months	Gingivitis
Iwasaki K. et al., 2016 [38]	n = 39 (≥60)/Periodontitis	*L. plantarum*	3 months	Periodontitis
Morales A. et al., 2016 [39]	n = 28 (≥40)/Periodontitis	*L. rhamnosus*	3 months	Periodontitis
Toiviainen A. et al., 2015 [40]	n = 62 (≥21)/Healthy	*L. rhamnosus*, *B. lactis*	1 month	Gingivitis

## Appendix A

**Table A1:** Summary of studies matching the inclusion criteria. SRP, scaling and root-planing. Y.o., years old. PPD, probing pocket depth. SPT, supportive periodontal therapy. PI, plaque index. GI, gingival index.

Authors/Year	Conclusions
Oda Y. et al., 2023 [26]	Daily consumed yogurt mixed with *L. rhamnosus* L8020 may stop the progression of periodontitis in subjects with intellectual disability.
Pørksen CJ. et al., 2023 [27]	Daily lozenge of *L. rhamnosus*, *L. paracasei* and arginine may reduce dental caries increment in children aged 5–9 y.o.
Ranjith A et al., 2022 [28]	Mouthwash with *L. rhamnosus*, *L. acidophilus*, *B. longum* and *S. boulardii* may be an adjunct to mechanical plaque removal in patients with stage II periodontitis.
Santana SI et al., 2022 [29]	Gel and lozenges containing *L. rhamnosus*, *L. paracasei* and *B. lactis* may promote additional clinical benefits to mechanical debridement in patients with peri-implant mucositis.
Morales A. et al., 2021 [30]	*L. rhamnosus* failed in enhancing additional clinical benefits in patients with stage III periodontitis compared to placebo.
Pudgar P. et al., 2021 [31]	The adjunctive use of probiotics containing *L. plantarum* and *L. brevis* in chronic periodontitis results in a lower gingival bleeding and a higher number of residual diseased sites when compared with SRP and placebo.
Yuki O. et al., 2019 [32]	Regular consumed yogurt mixed with *L. rhamnosus* L8020 may lower the risk of periodontal disease in subjects with intellectual disability.
Alanzi A. et al., 2018 [33]	Combination of *L. rhamnosus* and *B. lactis* may serve as a simple adjunct to standard oral care for promoting the oral health in adolescents aged 13–15 y.o.
Morales A. et al., 2018 [34]	Patients receiving SRP and sachets with *L. rhamnosus* showed a similar reduction in cultivable bacteria and clinical parameters as placebo or azithromycin.
Keller MK. et al., 2018 [35]	Tablets with *L. rhamnosus* and *L. curvatus* are able to reduce BOP and the flow of GCF, but not microbiological parameters in patients with moderate gingivitis.
Mongardini C. et al., 2017 [36]	The adjunctive use of *L. plantarum* and *L. brevis* does not significantly improve the clinical outcomes of professionally administered plaque removal in patients with peri-implant mucositis.
Montero E. et al., 2017 [37]	The adjunctive use of *L. plantarum*, L. *brevis* and *P. acidilactici* improve the subgingival microbiota in patients with gingivitis.
Iwasaki K. et al., 2016 [38]	Daily capsule containing *L. plantarum* may reduce PPD in patients during SPT.
Morales A. et al., 2016 [39]	The adjunct of sachets with *L. rhamnosus* to SRP lead to a greater reduction in PPD than placebo.
Toiviainen A. et al., 2015 [40]	Lozenges with *L. rhamnosus* and *B. lactis* could decrease both PI and GI, without changing the oral microbiota in healthy young adults.
